# Highly Sensitive and Rapid Identification of *Streptococcus agalactiae* Based on Multiple Cross Displacement Amplification Coupled With Lateral Flow Biosensor Assay

**DOI:** 10.3389/fmicb.2020.01926

**Published:** 2020-08-28

**Authors:** Xueqin Cheng, Zhiqian Dou, Jing Yang, Yulong Gu, Dexi Liu, Ling Xie, Tao Ren, Yan Liu, Zhifang Yu, Yijun Tang, Meifang Wang

**Affiliations:** ^1^Department of Respiratory and Critical Care Medicine, Taihe Hospital, Hubei University of Medicine, Shiyan, China; ^2^Hubei Key Laboratory of Embryonic Stem Cell Research, Taihe Hospital, Hubei University of Medicine, Shiyan, China; ^3^Department of Gynaecology and Obstetrics, Taihe Hospital, Hubei University of Medicine, Shiyan, China; ^4^Department of Pharmacy, Wuhan General Hospital of the Chinese People’s Liberation Army, Wuhan, China; ^5^Department of Clinical Laboratory, Taihe Hospital, Hubei University of Medicine, Shiyan, China; ^6^Department of Stomatology, Taihe Hospital, Hubei University of Medicine, Shiyan, China; ^7^Department of Dermatology, Taihe Hospital, Hubei University of Medicine, Shiyan, China

**Keywords:** *Streptococcus agalactiae*, multiple cross displacement amplification, lateral flow biosensor, MCDA-LFB, detection limit

## Abstract

*Streptococccus agalactiae* (*S. agalactiae*) is an important neonatal pathogen that is associated with mortality and morbidity. Therefore, we developed a rapid, accurate, and sensitive method based on multiple cross displacement amplification (MCDA) for the detection of the target pathogen. Four sets of MCDA primers were designed for targeting the *S. agalactiae*-specific *groEL* gene, and one set of MCDA primers with the optimum amplification efficiency was screened for establishing the *S. agalactiae*-MCDA assay. As a result, the newly-developed assay could be conducted at a fixed temperature (61°C) for only 30 min, eliminating the use of complex instruments. A portable and user-friendly nanoparticle-based lateral flow biosensor (LFB) assay was employed for reporting MCDA results within 2 min. Our results suggested that the detection limit of the *S. agalactiae*-MCDA-LFB assay is 300 fg per reaction, and no cross-reaction occurred with non-*S. agalactiae* strains. For 260 vaginal and rectal swabs, the detection rate of the MCDA-LFB assay was 7.7%, which was in accordance with the reference method of enrichment/qPCR, and higher by 4.6% than the CHROMagar culture. Moreover, the total procedure time of the MCDA-LFB assay was around 50 min, including sample collection, template preparation, MCDA reaction, and result reporting. Therefore, the MCDA-LFB assay is superior to enrichment/qPCR and CHROMagar culture and has great promise for point-of-care testing of *S. agalactiae* from vaginal and rectal swabs of pregnant women in resource-limited settings.

## Introduction

*Streptococccus agalactiae* is a common neonatal pathogen that can cause severe early-onset diseases, such as pneumonia, sepsis, and, less frequently, meningitis (Boyer and Gotoff, 1985; McGee et al., 2010). Due to the vertical transmission of *S. agalactiae* from carrier mothers to newborn infants, the maternal rectovaginal colonization becomes the most important risk element of early-onset disease. Therefore, timely detection of the maternal *S. agalactiae* carrier state can help clinicians to prevent the occurrence of vertical transmission during labor (Verani et al., 2010). However, little attention is given to this pathogen in developing countries, and only some third-class hospitals of major cities in China carried out *S. agalactiae* detection. According to some reported studies in mainland China, the maternal *S. agalactiae* colonization rate is about 3.7–14.52%, and the incidence rate of invasive *S. agalactiae* diseases in infants is estimated to be between 0.55 and 1.79 per 1,000 live births, with a fatality risk around 6.775%, which is still a substantial burden on developing countries such as China (Huang et al., 2019). In addition, a recent study reported that the incidence of early-onset disease is increasing annually in Guangzhou, China (Guan et al., 2015). Thus, prenatal screening and early diagnosis of *S. agalactiae* in actively laboring women in China are needed to help prevent the occurrence of severe disease in infants.

At present, an updated universal screening method based on a broth-enriched culture was recommended for pregnant women at 35–37 weeks of gestation, and intrapartum antibiotic treatment was advised for those who tested positive for *S. agalactiae* (Verani et al., 2010). However, this process is laborious and time-consuming, which is unhelpful for physicians who do not know how to treat infected infants (Konikkara et al., 2014). It is also suboptimally sensitive, and negative culture results have been observed in some women whose infants subsequently developed *S. agalactiae* infection (Lin et al., 2019). In addition, some pregnant women infected with *S. agalactiae* were misdiagnosed as having another *Streptococcus* species infection, as the test has low specificity (Goudarzi et al., 2015). Thus, a more rapid, accurate, and sensitive identification method for *S. agalactiae* is required to complement or replace the current culture method. To achieve this, a series of molecular techniques has been explored to detect *S. agalactiae*. Among these methods, qPCR is the most often developed molecular method that had been used for identifying *S. agalactiae* in many studies (Natarajan et al., 2006; Bergseng et al., 2007; Money et al., 2008; Martinez et al., 2010; Bourgeois-Nicolaos et al., 2013; Meehan et al., 2015; Tanaka et al., 2016). However, this method requires expensive laboratory equipment and highly-skilled professionals, which are not available in resource-limited settings. Moreover, the sensitivity of qPCR is controversial, as it varied from 62.5 to 98.5% compared to that of the bacterial culture method (Verani et al., 2010). The popular loop-mediated isothermal amplification (LAMP) assay has also been developed for detecting *S. agalactiae* (Kimura et al., 2013; Pu et al., 2019), and the LAMP products were usually analyzed by a turbidimeter or colorimetric indicator. However, the results obtained by a turbidimeter inevitably suffer from background interference, and the results are somewhat subjective when using colorimetric indicator with the naked eye, especially when using a limited quantity of DNA, usually leading to false-negative results. Thus, a more sensitive, objective, portable, and simple detection technique is needed.

Recently, a newly-developed molecular technique, termed multiple cross displacement amplification (MCDA), was shown to achieve specific and sensitive amplification of targeting genes at a constant temperature within 40 min (Wang et al., 2015). A total of 10 primers were designed in the MCDA assay, which in theory is more specific than nucleic acid amplification methods that use fewer primers, such as qPCR of two primers and LAMP of six primers. In order to rapidly determine whether the amplification occurred, an objective and sensitive LFB technique has been developed for specifically detecting the dual-labeled MCDA amplicons (Wang et al., 2017).

The aim of the present study was to develop a rapid, portable, user-friendly, and sensitive *S. agalactiae*-MCDA-LFB technique, which can assure reliable identification of *S. agalactiae*, and also effectively differentiate *S. agalactiae* from non-*S. agalactiae* strains. Here, we also detected the vaginal and rectal swabs with the MCDA-LFB assay to confirm its suitability for point-of-care rapid screening of *S. agalactiae*.

## Materials and Methods

### Reagents

TIANamp Bacteria DNA Kits were obtained from TIANGEN Biotech Co., Ltd. (Beijing, China). Lysozyme and agarose were obtained from Beyotime Biotechnology Co., Ltd. (Shanghai, China). The isothermal amplification kits, LFBs, and Malachite green (MG) were obtained from Beijing Hai Tai-Zheng Yuan Technology Co., Ltd. (Beijing, China). The length and width of the LFB are 6 and 4 mm, respectively, which is very portable. qPCR Mix was obtained from Takara Biomedical Technology Co., Ltd. (Beijing, China). The swabs were obtained from Copan Diagnostics, Inc. (Lombardy, Italy). Todd Hewitt medium, gentamicin, and nalidixic acid were obtained from Qingdao Hi-Tech Industrial Park Haibo Biotechnology Co., Ltd. (Qingdao, China).

### Genomic DNA Extraction

A total of 44 strains (Table 1) were used to evaluate the MCDA-LFB assay in this study, including 8 *S. agalactiae* strains and 36 non-*S. agalactiae* strains. Almost all the strains were collected from the Department of Microbial Laboratory, Taihe Hospital of Hubei University of Medicine, first identified by DL-96 systems using 96E ID Card, and further confirmed by conventional PCR targeting 16s rDNA and sequencing. Genomic DNA was extracted from the pure culture of each strain using a DNA Mini Kit according to the manufacturer’s instructions, and the extracted DNA was quantified with an ultraviolet spectrophotometer (NanoDrop One, Thermo, Beijing, China) at A260/280.

**Table 1:** Strains used in this study and the results of multiple cross displacement amplification (MCDA) assays.

Bacteria	Strain no./source	No. of strains	MCDA-LFB result^1^
*Streptococcus agalactiae*	THH-Sa001^2^	1	+
	Isolated strains	7	+
*Streptococcus pyogenes*	Isolated strain	2	−
*Streptococcus pneumoniae*	Isolated strain	2	−
*Streptococcus mitis*	Isolated strain	2	−
*Streptococcus salivarius*	Isolated strain	2	−
*Streptococcus sanguinis*	Isolated strain	2	−
*Streptococcus dysgalactiae*	Isolated strain	1	−
*Streptococcus gordonii*	Isolated strain	1	−
*Streptococcus sinensis*	Isolated strain	1	−
*Streptococcus contellatus*	Isolated strain	1	−
*Streptococcus anginosus*	Isolated strain	2	−
*Enterococcus faecalis*	Isolated strain	1	−
*Enterococcus faecium*	Isolated strain	1	−
*Enterococcus raffinosus*	Isolated strain	1	−
*Staphylococcus haemolyticus*	Isolated strain	1	−
*Staphylococcus hominis*	Isolated strain	1	−
*Staphylococcus aureus*	isolated strain	1	−
*Staphylococcus epidemidis*	isolated strain	1	−
*Staphylococcus saprophyticus*	Isolated strain	1	−
*Staphylococcus capitis*	Isolated strain	1	−
*Staphylococcus lugdunensis*	Isolated strain	1	−
*Micrococcus yunnanensis*	Isolated strain	1	−
*Klebsiella pneumoniae*	Isolated strain	1	−
*Citrobacter freumdii*	Isolated strain	1	−
*Bacillus mirabilis*	Isolated strain	1	−
*Enterobacter gergoviae*	Isolated strain	1	−
*Enterobacter cloacae*	Isolated strain	1	−
*Escherichia coli*	Isolated strain	1	−
*Lactobacillus jensenii*	Isolated strain	1	−
*Candida albicans*	Isolated strain	1	−
*Candida tropicalis*	Isolated strain	1	−

1 +, positive; −, negative.

2 THH, Taihe Hospital; Sa, *Streptococcus agalactiae*.

### Primer Design

**Figure 1:**
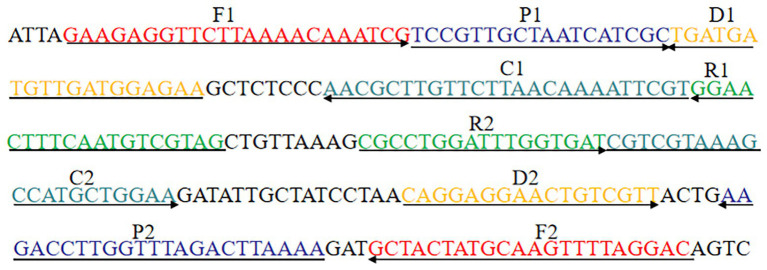
MCDA primer design for *groEL* gene.

In order to achieve the optimum amplification situation, a total of four sets (Supplementary Table S1) of MCDA primers targeting *groEL* gene (GenBank accession number EU003621) of *S. agalactiae* were designed by software Primer Premier 5.0 and PrimerExplorer V4. The *groEL* gene is a housekeeping gene of *S. agalactiae*, showing the greatest interspecies genetic diversity among 58 *Streptococcus* spp. (Glazunova et al., 2009). Each set of MCDA primers includes displacement primers F1 and F2, cross primers CP1 and CP2, and amplification primers C1, C2, D1, D2, R1, and R2. The primers’ screening was conducted, and the third set of MCDA primers, which showed better performance in rapidity and efficiency, was employed for establishing the *S. agalactiae*-MCDA assay. Then, 3D1 and 3R1 were 5'-labeled with fluorescein isothiocyanate (FITC) and biotin, respectively. The corresponding sequences of the third set of MCDA primers were demonstrated in Table 2, and the schematic design was shown in Figure 1. All MCDA primers were synthesized and purified by TsingKe Biotech Co., Ltd. (Beijing, China) at HPLC purification grade.

**Table 2:** The third set of primers used in this study.

Primer’s name	Sequences and modifications	Length	gene
groEL-3F1	5'-GAAGAGGTTCTTAAAACAAATCG-3'	21 nt^1^	groEL
groEL-3F2	5'-GTCCTAAAACTTGCATAGTAGC-3'	22 nt	
groEL-3CP1	5'-ACGAATTTTGTTAAGAACAAGCGTTTCCGTTGCTAATCATCGC-3'	43 mer^2^	
groEL-3CP2	5'-CGTCGTAAAGCCATGCTGGAATTTTAAGTCTAAACCAAGGTCTT-3'	44 mer	
groEL-3C1	5'-ACGAATTTTGTTAAGAACAAGCGTT-3'	25 nt	
groEL-3C2	5'-CGTCGTAAAGCCATGCTGGAA-3'	21 nt	
groEL-3D1	5'-TTCTCCATCAACATCATCA-3'	19 nt	
groEL-3D1^*^^3^	5'-FITC-TTCTCCATCAACATCATCA-3'	19 nt	
groEL-3D2	5'-CAGGAGGAACTGTCGTT-3'	17 nt	
groEL-3R1	5'-CTACGACATTGAAAGTTCC-3'	19 nt	
groEL-3R1^*^^4^	5'-Biotin-CTACGACATTGAAAGTTCC-3'	19 nt	
groEL-3R2	5'-CGCCTGGATTTGGTGAT-3'	17 nt	

1 nt, nucleotide.

2 mer, monomeric.

3 D1^*^, 5'-labeled with fluorescein isothiocyanate (FITC) when used in the MCDA-lateral flow biosensor (LFB) assay.

4 R1^*^, 5'-labeled with biotin when used in the MCDA-LFB assay.

### MCDA Reactions

A 25 μl *groEL*-MCDA reaction mixture containing 0.4 μM displacement primers F1 and F2, 0.8 μM amplification primers C1, C2, D1^*^, D2, R1^*^, and R2, 1.6 μM cross primer CP1 and CP2, 1 μl Bst DNA polymerase (10 U), 12.5 μl 2X Reaction Buffer, 1.5 μl MG, and 1 μl DNA template was conducted at a constant temperature.

We used three detection methods to analyze *groEL*-MCDA products, including MG, gel electrophoresis, and LFB. With MG, the color of the reaction mixture turning from green to bright green demonstrates positive results, but turning colorless shows negative results. With 2% gel electrophoresis, a ladder band appeared for *S. agalactiae*, but no ladder bands appeared for non-*S. agalactiae* strains. When MCDA products were analyzed by LFB, both control line (CL) and test line (TL) were visual for *S. agalactiae*, and only CL was visual for non-*S. agalactiae* strains.

*S. agalactiae* strain THH-Sa001 was used as a reference strain in this study. *Streptococcus pyogenes*, *Streptococcus pneumoniae*, and distilled water (DW) were used as negative control, negative control, and blank control, respectively. Then, monitoring techniques, including MG, gel electrophoresis, and LFB, were used to confirm the feasibility of *groEL*-MCDA primers.

The MCDA reaction with 3 pg *S. agalactiae* strain THH-Sa001 DNA was incubated at eight different temperatures (ranging from 60 to 67°C at 1°C interval) for 40 min, to determine the optimum amplification temperature. Seven different DNA concentrations of *S. agalactiae* strain THH-Sa001 (3 ng μl^−1^ to 3 fg μl^−1^) were amplified for 10, 20, 30, and 40 min to determine the optimum detection time.

### Sensitivity and Specificity of LFB Assay for Detecting MCDA Products

Genomic DNA of *S. agalactiae* strain THH-Sa001 diluted from 3 ng μl^−1^ to 3 fg μl^−1^ was added to the MCDA reaction and conducted at a constant temperature to determine the sensitivity of the *S. agalactiae*-MCDA-LFB assay. DNA extracted from *S. agalactiae*, other *Streptococcus* species, and non-*Streptococcus* species was used in this study to determine the specificity of the *S. agalactiae*-MCDA-LFB assay.

### *S. agalactiae* Culture

The collected vaginal and rectal swabs were inoculated on CHROMagar plates and incubated at a 37°C thermostat with 5% CO2. After 24 h incubation, the purple colonies were sub-cultured in blood agar plates and Christie-Atkins-Munch-Peterson (CAMP)-tested. The colonies that CAMP-tested positive were further confirmed by conventional PCR and sequencing.

### *S. agalactiae*-MCDA-LFB Assays Applied in Vaginal and Rectal Swabs

A total of 260 vaginal and rectal swabs were collected from pregnant women with a gestational age ≥24 weeks, who did not use any antibiotics in the 30 days prior to specimen collection. All collected swabs were stored in Copan’s Transport Medium. Each swab head was rubbed in 80 μl DW, and 20 μl was inoculated on CHROMagar plates. The other 20 μl was added to Todd Hewitt selective medium for enriched culture about 24 h, and the DNA was prepared, as previously described (Vieira et al., 2019). The remaining liquid was heated at 100°C for 15 min, and 2 μl of which was directly detected by the MCDA-LFB assay. The qPCR primer sequences used were 5'-TTTCACCAGCTGTATTAGAAGTA-3' and 5'-GTTCCCTGAACATTATCTTTGAT-3' (Vieira et al., 2019).

qPCR reactions were conducted in a total of 20 μl volume containing 0.4 μM forward and reverse primer each, 10 μl TB Green Fast qPCR Mix, and 2 μl DNA. Reaction conditions were set at 95°C for 30 s, followed by 40 cycles of 95°C for 5 s and 60°C for 34 s.

## Results

### Screening of *S. agalactiae*-MCDA Primers

**Figure 2:**
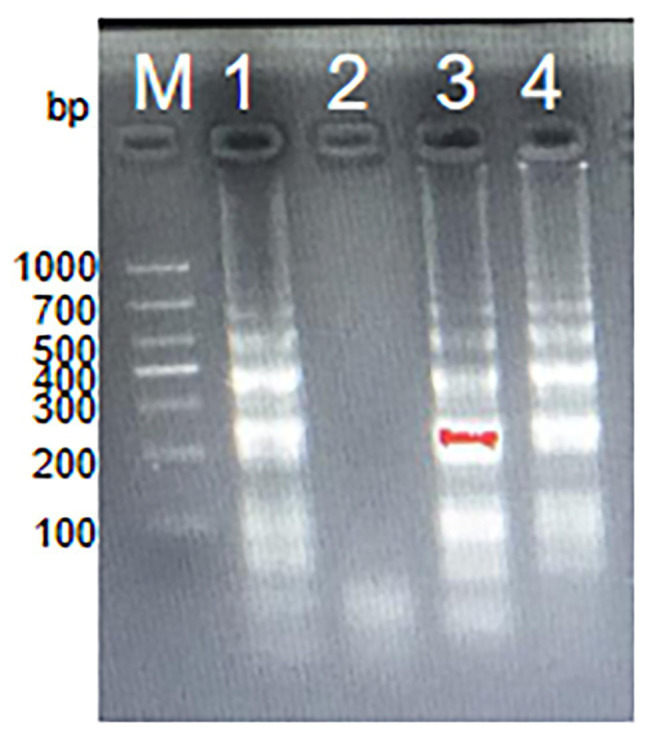
The amplification products using four sets of MCDA primers. Agarose gel electrophoresis was used to evaluate the amplification efficiency of four sets of MCDA primers. Lane M, DNA maker DL1000; Lane 1, the first set of MCDA primers; Lane 2, the second set of MCDA primers; Lane 3, the third set of MCDA primers; Lane 4, the fourth set of MCDA primers.

A total of four sets of MCDA primers designed in this study were used to evaluate the amplification efficiency. Gel electrophoresis was used to analyze MCDA products. As observed in Figure 2, no amplicons were observed for the second set of MCDA primers. The same bright ladder bands were observed for the first set of MCDA primers and the fourth set of MCDA primers. The brightest ladder bands were observed for the third set of MCDA primers. These results demonstrated that the third set of MCDA primers displayed a better performance in amplification efficiency. Thus, the third set of MCDA primers was selected for subsequent experiments.

### Confirmation of MCDA Primers Targeting on *groEL* Gene of *S. agalactiae*

**Figure 3:**
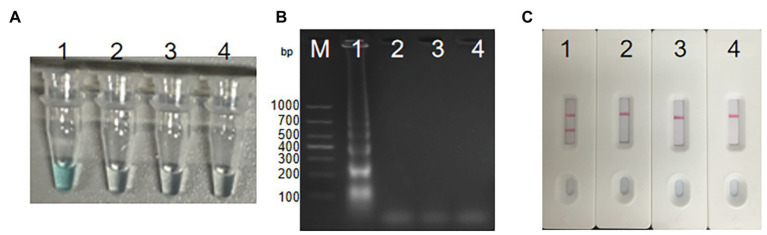
Detection for *S. agalactiae*-MCDA products. MCDA amplicons were analyzed by three methods, including Malachite green (MG) **(A)**, 2% agarose gel electrophoresis **(B)**, and LFB **(C)**. Lane M, DNA maker DL1000. Tube/lane/biosensor 1–4 respectively represent positive group (*S. agalactiae* strain THH-Sa001), negative group (*Streptococcus pyogenes* and *Streptococcus pneumoniae*), and blank group (DW). TL, test line; CL, control line.

In order to analyze the feasibility of the third set of MCDA primers, a total of three groups of DNA were amplified at 62°C for 1 h, including the positive group of *S. agalactiae* strain THH-Sa001, the negative group of *S. pyogenes* or *S. pneumoniae*, and the blank group of DW. Then, three mature measurement technologies were used to detect MCDA products, such as MG, gel electrophoresis, and LFB. As observed in Figure 3, DNA from *S. agalactiae* produced positive amplicons, and the reaction system of tube 1 turned bright green, but tube 2 of *S. pyogenes*, tube 3 of *S. pneumoniae*, and tube 4 of DW turned colorless, reporting as negative. By LFB, two red lines (CL and TL) were visual for *S. agalactiae*, but only one red line (CL) was visual for negative controls and the blank control. With 2% gel electrophoresis, a ladder band was observed for *S. agalactiae*, but no ladder bands were observed for negative controls or the blank control. Thus, these results demonstrated that the third set of MCDA primers screened here was a good candidate for the detection of *S. agalactiae*.

### The Optimum Amplification Temperature of *groEL*-MCDA Primers

**Figure 4:**
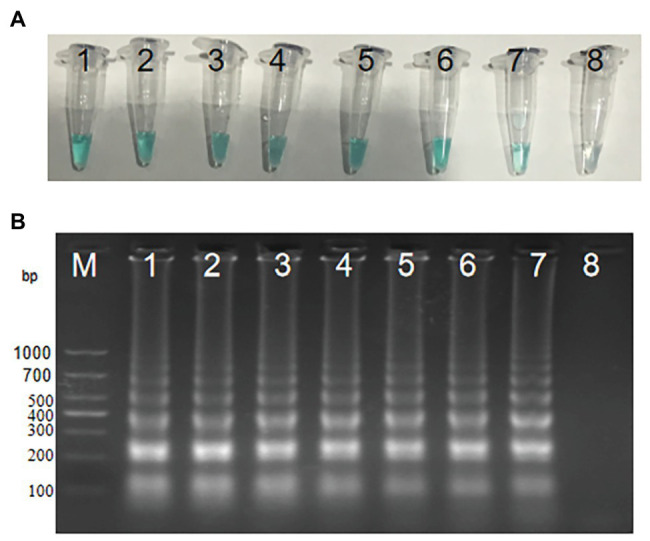
Optimal amplification temperature for *S. agalactiae*-MCDA primers. Two methods, including MG **(A)** and agarose gel electrophoresis **(B)**, were used to evaluate the amplification situation of the *S. agalactiae*-MCDA assay. Tubes/agarose gel electrophoresis 1–8 respectively represent 60, 61, 62, 63, 64, 65, 66, and 67°C.

In order to explore the optimum amplification temperature, we conducted MCDA reactions with 3 pg DNA at eight different temperatures ranging from 60 to 67°C with 1°C interval for 40 min. As shown in Figures 4A,B, MCDA reactions conducted at 60–66°C turned bright green, detectable by the naked eye, but were almost colorless at 67°C. MCDA products analyzed by 2% gel electrophoresis showed that 60–61°C was the optimum amplification situation of *groEL* gene of *S. agalactiae*. Thus, 61°C was selected for the following experiment.

### The Minimum Detection Limit of MCDA-LFB Assays for *S. agalactiae*

**Figure 5:**
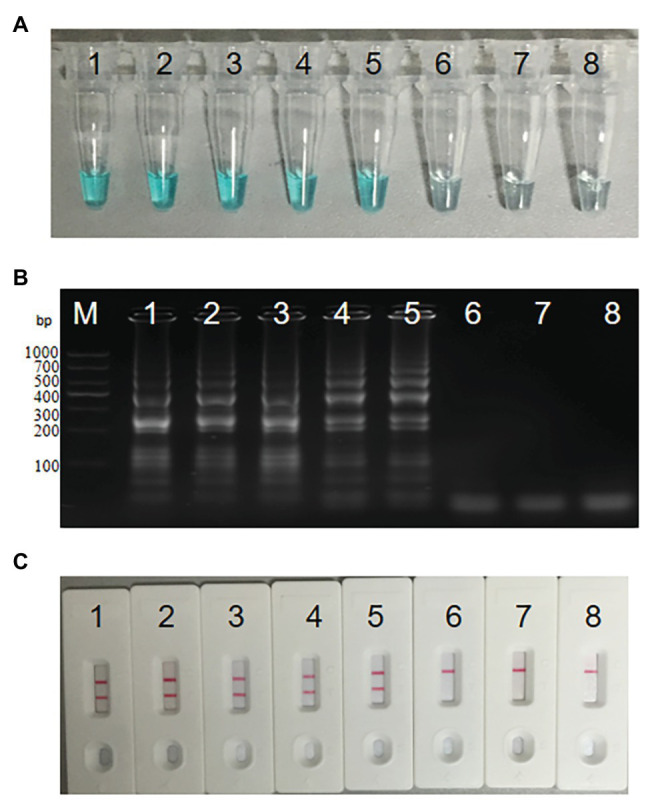
Sensitivity analysis of *S. agalactiae*-MCDA-LFB assay. Three diagnosis techniques, including colorimetric indicator **(A)**, gel electrophoresis **(B)**, and LFB **(C)**, were used for analysis of MCDA amplicons. Tubes **(A)**/lanes **(B)**/biosensors **(C)** 1–8 respectively represent *S. agalactiae* strain THH-Sa001 DNA levels of 3 ng, 300 pg, 30 pg, 3 pg, 300 fg, 30 fg, and 3 fg per reaction, and a blank control (DW).

We diluted *S. agalactiae* strain THH-Sa001 DNA into seven concentrations (3 ng μl^−1^ to 3 fg μl^−1^), and then added the above diluted DNA of 1 μl into the MCDA system and incubated it at 61°C for 1 h,. As observed in Figure 5, two red lines appeared at the LFB for as little as 300 fg (946 CFU per reaction) of the DNA template. The detection limit obtained using MG detection or the gel electrophoresis test for *S. agalactiae* was also 300 fg, which was the same as that of the LFB assay. Thus, the detection limit of the MCDA-LFB assay for *S. agalactiae* was 300 fg.

### The Optimum Time of *groEL*-MCDA-LFB Assays

**Figure 6:**
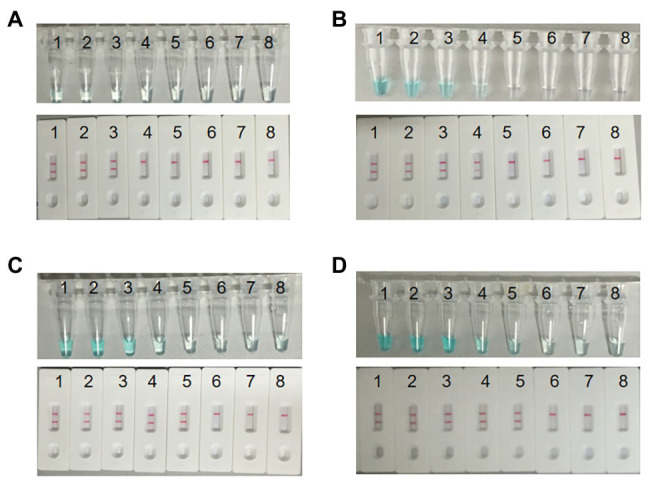
Optimal detection time required for *S. agalactiae* MCDA-LFB assay. Four reaction times (**A**, 10 min; **B**, 20 min; **C**, 30 min; **D**, 40 min) were selected to determine the optimal detection time at 61°C. Biosensors 1–8 represent *S. agalactiae* strain THH-Sa001 DNA levels of 3 ng, 300 pg, 30 pg, 3 pg, 300 fg, 30 fg, and 3 fg per reaction, and a blank control (DW), respectively. The minimum detection limit was observed when MCDA lasted for 30 min **(C)**.

As observed in Figure 6, when the reaction time was 10 min, the initial DNA amount of 30 pg above in MCDA reactions can produce FITC‐ and biotin-attached duplex amplicons, and two red lines appeared at the LFB. When using MG, the color of the reaction mixture of the initial DNA amount of 3 ng below was almost colorless at the end point of amplification, reporting negative results. When the reaction time was delayed to 20 min, the LFB can detect the MCDA products of the initial DNA amount of 3 pg above. However, MG is faced with the same problem that takes place in 10 min, when the color of the reaction solution was almost colorless at the initial DNA amount of 3 pg and 3 pg below. When the reaction time continues for 30 and 40 min, the LFB can detect the MCDA products of the initial DNA amount of 300 fg above, while MG can detect the MCDA products of the initial DNA amount of 3 pg above for 30 min and 300 fg above for 40 min. Thus, 30 min was the optimum time for the MCDA-LFB assay.

### The Specificity of MCDA-LFB Assays for *S. agalactiae*

**Figure 7:**
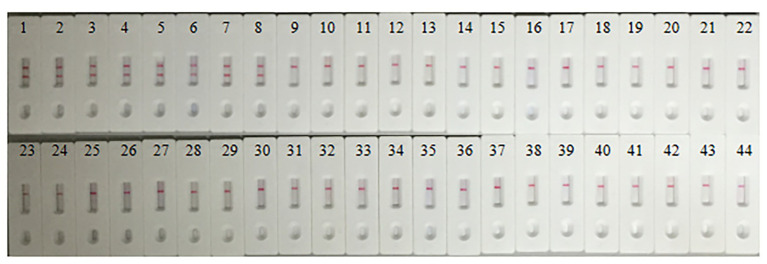
Specificity of *S. agalactiae*-MCDA-LFB assays. A total of 44 strains were used to evaluate the specificity of MCDA-LFB assays. 1, *Streptococcus agalactiae* strain THH-Sa001; 2–8, *Streptococcus agalactiae*; 9–10, *Streptococcus pyogenes*; 11–12, *Streptococcus pneumoniae*; 13–14, *Streptococcus mitis*; 15–16, *Streptococcus salivarius*; 17–18, *Streptococcus sanguinis*; 19, *Streptococcus dysgalactiae*; 20, *Streptococcus gordonii*; 21, *Streptococcus sinesis*; 22, *Streptococcus contellatus*; 23–24, *Streptococcus anginosus*; 25, *Enterococcus faecalis*; 26, *Enterococcus faecium*; 27, *Enterococcus raffinosus*; 28, *Staphylococcus haemolyticus*; 29, *Staphylococcus hominis*; 30, *Staphylococcus aureus*; 31, *Staphylococcus epidemidis*; 32, *Staphylococcus saprophyticus*; 33, *Staphylococcus capitis*; 34, *Staphylococcus lugdunensis*; 35, *Micrococcus yunnanensis*; 36, *klebsiella pneumoniae*; 37, *Citrobacter freumdii*; 38, *Bacillus mirabilis*; 39, *Enterobacter gergoviae*; 40, *Enterobacter cloacae*; 41, *Escherichia coli*; 42, *Lactobacillus jensenii*; 43, *Candida albicans*; 44, *Candida tropicalis*.

A total of 44 strains listed in Table 1 were used to determine the specificity of the MCDA-LFB assay for *S. agalactiae*, including 8 *S. agalactiae* strains, 19 other *Streptococccus* species, and 17 non-*Streptococccus* strains causing vaginal infection. As a result, except for non-*S. agalactiae* strains, all *S. agalactiae* strains produced MCDA amplicons, and two red lines were visual at the LFB (Figure 7). These results showed that the MCDA-LFB assay was highly selective for *S. agalactiae*.

### MCDA-LFB Assays for Rapid Detection of *S. agalactiae* in Vaginal and Rectal Swabs

**Figure 8:**
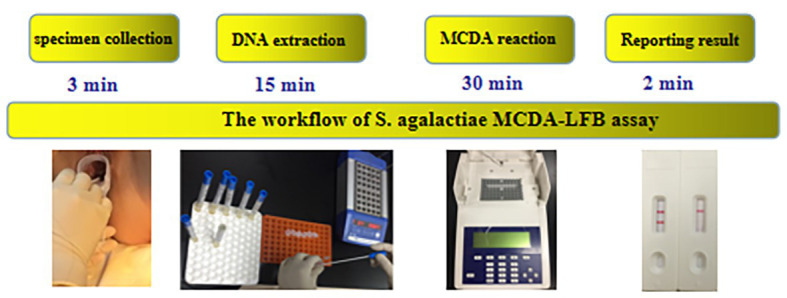
The workflow of *S. agalactiae* MCDA-LFB assays. Four steps were required for completing the *S. agalactiae* MCDA-LFB test, including specimen collection (3 min), DNA extraction (15 min), MCDA reaction (30 min), and result reporting (2 min).

In order to confirm the clinical application value, the successfully developed MCDA-LFB assay was used to test 260 vaginal and rectal swabs, which were also detected by CHROMagar culture and enrichment/qPCR. The result showed that 20 samples tested positive by the MCDA-LFB assay, which was the same amount as that of enrichment/qPCR. Eight samples tested positive by CHROMagar culture (Table 3). Compared to enrichment/qPCR, there were no very major errors, major errors, or minor errors. Compared to CHROMagar culture, there were no very major errors or minor errors, and the major error was 4.6%. The MCDA-LFB assay only requires 50 min for detecting each clinical sample, which is shorter than the 25.6 h required for enrichment/qPCR and the 24 h for CHROMagar culture. Thus, the MCDA-LFB assay was superior to enrichment/qPCR and CHROMagar culture, and would be a valuable laboratory test for vaginal and rectal swabs. The graphical diagram from the beginning of the specimen collection until the interpretation of the MCDA-LFB assay is shown in Figure 8.

**Table 3:** The MCDA-LFB assay compared to CHROMagar culture and enrichment/qPCR in detection *S. agalactiae* from vaginal and rectal swabs.

MCDA-LFB	CHROMagar culture		Enrichment/qPCR	
	Positive	Negative	Positive	Negative
Positive	8	12	20	0
Negative	0	240	0	240
Very major error	0		0	
Major error	4.6%		0	
The minor error	0		0	

## Discussion

With the popularization of broth-enriched culture and intrapartum antibiotic prophylaxis, the incidence of early-onset disease and the mortality rate in the western world significantly declined (Verani, et al., 2010). However, up to 13.4% of pregnant women in the USA have not accepted routine screening due to various reasons, such as fear of preterm (Van Dyke et al., 2009; Goins et al., 2010). In China, there are no standard guidelines for detecting *S. agalactiae*, leading to many pregnant women going without screening. Moreover, a 2009 study demonstrated that 70% of early-onset diseases appeared in the asymptomatic colonization mothers who tested as unknown or negative at a prenatal screening during 35–37 weeks’ gestation (Tazi et al., 2009). Thus, a rapid, convenient, sensitive, and low-cost detecting method for *S. agalactiae* is in an urgent need to meet the current clinical diagnosis requirements.

This study, then, was directed against the disadvantages of bacterial culture and the present molecular tests and aimed to develop a new detecting method, which had been successfully applied to the specific detection of some bacterium, such as *Staphylococcus aureus*, *Acinetobacter baumannii*, and *Mycoplasma pneumoniae* (Wang et al., 2018, 2019; Cheng et al., 2019). The MCDA assay is very economical, at a cost of only US$ 3.5. The time it takes here was also shortened, taking only 30 min, which is much faster than the 2 h reaction time of qPCR and 48–72 h reaction time of broth-enriched culture (Carrillo-Avila et al., 2018). The only instrument required for the MCDA assay is a thermostatic apparatus, which is easily achieved in remote districts, unlike qPCR that depends on expensive thermal cycling equipment or a CO2 incubator that is required for the culture method. Thus, the MCDA assay has many advantages over the qPCR and culture method, and might become a potential tool for detecting *S. agalactiae* in either primary hospitals or large-scale hospitals in the future.

The minimum detection limit of the *S. agalactiae*-MCDA-LFB assay is 300 fg, which is 333-fold more sensitive than the *S. agalactiae*-PCR assay, the detection threshold of which was 100 pg (Pu et al., 2019). Apart from its excellent sensitivity, the MCDA-LFB assay also has high specificity that can clearly differentiate *S. agalactiae* from other *Streptococcus* and non-*Streptococcus* species. The prevalence of *S. agalactiae* by the MCDA-LFB assay was 7.7%, which was in accordance with the standard method of enrichment/qPCR. However, this standard method takes about 25.6 h to obtain the final result, which is too long time for preterm labor and women in labor. The newly-developed MCDA-LFB assay here only takes about 50 min for each clinical specimen, which significantly shortens the turnaround time and simultaneously guarantees high sensitivity. The prevalence of *S. agalactiae* by CHROMagar culture was 3.1%, which was similar to the results previously reported (Ji et al., 2019). This may be due to the fact that *Staphylococcus* species and *Enterococcus* species also displayed purple colonies on the CHROMagar plates, which may lead to the missed detection of *S. agalactiae*. All these results demonstrated that the MCDA-LFB assay is a rapid, sensitive, and specific technique for detecting *S. agalactiae* directly from vaginal and rectal swabs.

The present common methods used for detecting isothermal amplification products include colorimetric indicators, gel electrophoresis, and turbidity, with colorimetric indicators becoming the most popular detecting method due to the lack of sophisticated equipment required. The cost of MG per test is US$ 1.4, which is slightly cheaper than the US$ 2 of an LFB. However, the results obtained by MG are subjective and are not as sensitive as LFB. When the number of amplicons in the reaction mixture is very low, it is not easy to differentiate weak-positive results from negative results using the color of the reaction mixture, leading to false-negative results in some cases. Moreover, the MG used in this study is a DNA binding dye whose fluorescence intensity will strongly increase in the presence of any double-stranded DNA, such as primer dimers, without specific combinations, which may lead to false-positive results. These false results may further lead to unnecessary intrapartum antibiotic prophylaxis and an increase in hospitalization expenses, meaning the final cost for MG may be even higher than LFB. Then, a very objective, specific, and simple LFB assay was designed, which was based on anti-FITC. The LFB test line captured FITC on the duplex and streptavidin on the nanoparticles combined with biotin on the duplex, and an obvious red line was visible on the location of the test line for targeting pathogens without ambiguity. The new assay is also very user-friendly and rapid, with results obtained within only 2 min. The only disadvantage of the LFB is that the lid must be opened for detection, increasing the chance of aerosol pollution. However, this contamination could be successfully avoided by adding antarctic thermal sensitive uracil-DNA-glycosylase (AUDG) enzyme into the MCDA reaction mixture before the initiation of amplification. Thus, comprehensive consideration of the advantages and disadvantages of MG and the LFB assay shows that the LFB would be a better choice for detecting *S. agalactiae*-MCDA products.

## Conclusion

In the present study, the high-efficiency MCDA technique, coupled with the portable LFB assay, was successfully developed for *S. agalactiae* detection based on the *groEL* gene. The whole test of the *S. agalactiae*-MCDA-LFB assay can be completed within 50 min. The new method also has excellent sensitivity and specificity. All these merits make it a useful diagnostic tool for point-of-care testing of *S. agalactiae* from vaginal and rectal swabs. Whether the MCDA-LFB method could be applied in detecting various other types of *S. agalactiae*-infected patient clinical specimens may lead to the wide application of this rapid, cost-effective, and easy-to-use point-of-care test for pathogens in resource-limited districts.

## Data Availability Statement

All datasets presented in this study are included in the article/Supplementary Material.

## Ethics Statement

The studies involving human participants were reviewed and approved by the Ethics Committee of Shiyan Taihe Hospital (number: 2020KS009). The patients/participants provided their written informed consent to participate in this study.

## Author Contributions

XC and JY designed the study. ZD collected clinical specimens. DL and LX revised the language. YG performed the culture experiments of *S. agalactiae* and CAMP test. TR, YL, and ZY carried out qPCR experiments and DNA extraction. XC and ZD conducted the MCDA-LFB experiments, wrote the manuscript, and contributed equally to this article. YT and MW were responsible for the overall supervision of the study and funded this study. All authors contributed to the article and approved the submitted version.

### Conflict of Interest

The authors declare that the research was conducted in the absence of any commercial or financial relationships that could be construed as a potential conflict of interest.

## References

[ref24] Bergseng H. Bevanger L. Rygg M. Bergh K. (2007). Real-time PCR targeting the sip gene for detection of group B *Streptococcus* colonization in pregnant women at delivery. J. Med. Microbiol. 56, 223–228. 10.1099/jmm.0.46731-0, PMID: 17244804

[ref25] Bourgeois-Nicolaos N. Cordier A. G. Guillet-Caruba C. Casanova F. Benachi A. Doucet-Populaire F. (2013). Evaluation of the Cepheid Xpert GBS assay for rapid detection of group B streptococci in amniotic fluids from pregnant women with premature rupture of membranes. J. Clin. Microbiol. 51, 1305–1306. 10.1128/JCM.03356-12, PMID: 23390279 PMC3666785

[ref1] Boyer K. M. Gotoff S. P. (1985). Strategies for chemoprophylaxis of GBS early-onset infections. Antibiot. Chemother. 35, 267–280. 10.1159/000410380, PMID: 3931544

[ref2] Carrillo-Avila J. A. Gutierrez-Fernandez J. Gonzalez-Espin A. I. Garcia-Trivino E. Gimenez-Lirola L. G. (2018). Comparison of qPCR and culture methods for group B *Streptococcus* colonization detection in pregnant women: evaluation of a new qPCR assay. BMC Infect. Dis. 18:305. 10.1186/s12879-018-3208-4, PMID: 29976153 PMC6034337

[ref3] Cheng X. Yang J. Wang M. Wu P. Du Q. He J.. (2019). Visual and rapid detection of *Acinetobacter baumannii* by a multiple cross displacement amplification combined with nanoparticles-based biosensor assay. AMB Express 9:30. 10.1186/s13568-019-0754-0, PMID: 30806854 PMC6391507

[ref4] Glazunova O. O. Raoult D. Roux V. (2009). Partial sequence comparison of the rpoB, sodA, groEL and gyrB genes within the genus *Streptococcus*. Int. J. Syst. Evol. Microbiol. 59, 2317–2322. 10.1099/ijs.0.005488-0, PMID: 19620365

[ref5] Goins W. P. Talbot T. R. Schaffner W. Edwards K. M. Craig A. S. Schrag S. J.. (2010). Adherence to perinatal group B streptococcal prevention guidelines. Obstet. Gynecol. 115, 1217–1224. 10.1097/AOG.0b013e3181dd916f, PMID: 20502293 PMC3773817

[ref6] Goudarzi G. Ghafarzadeh M. Shakib P. Anbari K. (2015). Culture and real-time PCR based maternal screening and antibiotic susceptibility for group B *Streptococcus*: an Iranian experience. Global J. Health Sci. 7, 233–239. 10.5539/gjhs.v7n6p233, PMID: 26153188 PMC4803879

[ref7] Guan X. Liu H. Zhong H. Peng X. Pang S. Huang L. (2015). Incidence and clinical characteristics of invasive group B streptococcal disease of young infants in Guangzhou. J. Appl. Clin. Pediatr. 31, 765–768.

[ref8] Huang J. Lin X. Z. Zhu Y. Chen C. (2019). Epidemiology of group B streptococcal infection in pregnant women and diseased infants in mainland China. Pediatr. Neonatol. 60, 487–495. 10.1016/j.pedneo.2019.07.001, PMID: 31445795

[ref9] Ji Y. Zhao C. Ma X. X. Peppelenbosch M. P. Ma Z. Pan Q. (2019). Outcome of a screening program for the prevention of neonatal early-onset group B *Streptococcus* infection: a population-based cohort study in Inner Mongolia, China. J. Med. Microbiol. 68, 803–811. 10.1099/jmm.0.000976, PMID: 30994439

[ref10] Kimura K. Yanagisawa H. Wachino J. Shibayama K. Arakawa Y. (2013). Rapid and reliable loop-mediated isothermal amplification method for detecting *Streptococcus agalactiae*. Jpn. J. Infect. Dis. 66, 546–548. 10.7883/yoken.66.546, PMID: 24270149

[ref11] Konikkara K. P. Baliga S. Shenoy S. Bharati B. (2014). Evaluation of culture, antigen detection and polymerase chain reaction for detection of vaginal colonization of group B *Streptococcus* (GBS) in pregnant women. J. Clin. Diagn. Res. 8, 47–49. 10.7860/JCDR/2014/6675.4004, PMID: 24701479 PMC3972595

[ref12] Lin Y. Ye J. Luo M. Hu B. Wu D. Wen J.. (2019). Group B *Streptococcus* DNA copy numbers measured by digital PCR correlates with perinatal outcomes. Anal. Chem. 91, 9466–9471. 10.1021/acs.analchem.8b05872, PMID: 31269399

[ref13] Martinez D. T. B. Stan C. M. Boulvain M. Renzi G. Francois P. Irion O.. (2010). Development of a rapid PCR assay for screening of maternal colonization by group B *Streptococcus* and neonatal invasive *Escherichia coli* during labor. Gynecol. Obstet. Investig. 70, 250–255. 10.1159/000314014, PMID: 21051844

[ref14] McGee L. Schrag S. J. Verani J. R. (2010). Prevention of perinatal group B streptococcal disease: revised guidelines from CDC, 2010. Atlanta, GA: Department of Health and Human Services, Centers for Disease Control and Prevention. 21088663

[ref26] Meehan M. Cafferkey M. Corcoran S. Foran A. Hapnes N. LeBlanc D.. (2015). Real-time polymerase chain reaction and culture in the diagnosis of invasive group B streptococcal disease in infants: a retrospective study. Eur. J. Clin. Microbiol. Infect. Dis. 34, 2413–2420. 10.1007/s10096-015-2496-5, PMID: 26433745

[ref27] Money D. Dobson S. Cole L. Karacabeyli E. Blondel-Hill E. Milner R.. (2008). An evaluation of a rapid real time polymerase chain reaction assay for detection of group B *Streptococcus* as part of a neonatal group B *Streptococcus* prevention strategy. J. Obstet. Gynaecol. Can. 30, 770–775. 10.1016/S1701-2163(16)32940-1, PMID: 18845045

[ref28] Natarajan G. Johnson Y. R. Zhang F. Chen K. M. Worsham M. J. (2006). Real-time polymerase chain reaction for the rapid detection of group B streptococcal colonization in neonates. Pediatrics 118, 14–22. 10.1542/peds.2005-1594, PMID: 16818544 PMC1513630

[ref15] Pu W. Wang Y. Yang N. Guo G. Li H. Li Q.. (2019). Investigation of *Streptococcus agalactiae* using pcsB-based LAMP in milk, tilapia and vaginal swabs in Haikou. J. Appl. Microbiol. 128, 784–793. 10.1111/jam.14501, PMID: 31651063

[ref29] Tanaka K. Iwashita M. Matsushima M. Wachi Y. Izawa T. Sakai K.. (2016). Intrapartum group B *Streptococcus* screening using real-time polymerase chain reaction in Japanese population. J. Matern. Fetal Neonatal Med. 29, 130–134. 10.3109/14767058.2014.989496, PMID: 25471089

[ref16] Tazi A. Doloy A. Reglier-Poupet H. Hemet M. E. Raymond J. Poyart C. (2009). Evaluation of the new chromogenic medium StrepB select for screening of group B *Streptococcus* in pregnant women. Pathol. Biol. 57, 225–228. 10.1016/j.patbio.2008.09.002, PMID: 19008052

[ref17] Van Dyke M. K. Phares C. R. Lynfield R. Thomas A. R. Arnold K. E. Craig A. S.. (2009). Evaluation of universal antenatal screening for group B *Streptococcus*. N. Engl. J. Med. 360, 2626–2636. 10.1056/NEJMoa0806820, PMID: 19535801

[ref18] Verani J. R. McGee L. Schrag S. J. (2010). Prevention of perinatal group B streptococcal disease--revised guidelines from CDC, 2010. MMWR Recomm. Rep. 59, 1–36. PMID: 21088663

[ref19] Vieira L. L. Perez A. V. Machado M. M. Kayser M. L. Vettori D. V. Alegretti A. P.. (2019). Group B *Streptococcus* detection in pregnant women: comparison of qPCR assay, culture, and the Xpert GBS rapid test. BMC Pregnancy Childbirth 19:532. 10.1186/s12884-019-2681-0, PMID: 31888631 PMC6937909

[ref20] Wang Y. Wang Y. Ma A. J. Li D. X. Luo L. J. Liu D. X.. (2015). Rapid and sensitive isothermal detection of nucleic-acid sequence by multiple cross displacement amplification. Sci. Rep. 5:11902. 10.1038/srep11902, PMID: 26154567 PMC4648395

[ref21] Wang Y. Wang Y. Quan S. Jiao W. Li J. Sun L.. (2019). Establishment and application of a multiple cross displacement amplification coupled with nanoparticle-based lateral flow biosensor assay for detection of *Mycoplasma pneumoniae*. Front. Cell. Infect. Microbiol. 9:325. 10.3389/fcimb.2019.00325, PMID: 31608243 PMC6767991

[ref22] Wang Y. Wang Y. Zhang L. Xu J. Ye C. (2017). Visual and multiplex detection of nucleic acid sequence by multiple cross displacement amplification coupled with gold nanoparticle-based lateral flow biosensor. Sensors Actuators B Chem. 241, 1283–1293. 10.1016/j.snb.2016.10.001

[ref23] Wang Y. Yan W. Fu S. Hu S. Wang Y. Xu J.. (2018). Multiple cross displacement amplification coupled with nanoparticles-based lateral flow biosensor for detection of *Staphylococcus aureus* and identification of methicillin-resistant *S. aureus*. Front. Microbiol. 9:907. 10.3389/fmicb.2018.03354, PMID: 29867818 PMC5954800

